# Supplementary material for: Mechanisms governing the pioneering and redistribution capabilities of the non-classical pioneer PU.1
Source: Nat Commun. 2020 Jan 21;11:402. doi: 10.1038/s41467-019-13960-2 (PMC6972792; doi:10.1038/s41467-019-13960-2)
Supplement: Supplementary file 7 — Source data [file 41467_2019_13960_MOESM7_ESM.zip › Source_Data/FigureS2/FigureS2B_MotifScanOutput/KmeansCluster8/homerResults/motif34.similar.html]

motif34

## Information for motif34

A
G
T
C
A
G
T
C
A
G
T
C
C
T
G
A
A
C
G
T
C
T
A
G
C
G
T
A
G
A
C
T
G
A
T
C
A
G
T
C
C
G
T
A
C
G
T
A
C
G
A
T
A
G
T
C
  
Reverse Opposite:  

C
T
A
G
C
G
T
A
G
C
A
T
A
C
G
T
C
T
A
G
C
T
A
G
C
T
G
A
A
C
G
T
A
G
T
C
C
G
T
A
A
G
C
T
C
T
A
G
C
T
A
G
C
T
A
G
  

|  |  |
| --- | --- |
| p-value: | 1e-17 |
| log p-value: | -4.047e+01 |
| Information Content per bp: | 1.817 |
| Number of Target Sequences with motif | 27.0 |
| Percentage of Target Sequences with motif | 1.79% |
| Number of Background Sequences with motif | 69.8 |
| Percentage of Background Sequences with motif | 0.15% |
| Average Position of motif in Targets | 115.7 +/- 75.2bp |
| Average Position of motif in Background | 118.3 +/- 56.0bp |
| Strand Bias (log2 ratio + to - strand density) | 0.5 |
| Multiplicity (# of sites on avg that occur together) | 1.00 |
| Motif File: | file (matrix) reverse opposite |

### Similar de novo motifs found

|  |  |  |  |  |  |  |  |
| --- | --- | --- | --- | --- | --- | --- | --- |
| Rank | Match Score | Redundant Motif | P-value | log P-value | % of Targets | % of Background | Motif file |
| 1 | 0.898 | C T G A T C G A G C A T A C G T C T A G C T A G C G T A C G A T G T A C C G T A A G C T C A T G C T A G | 1e-15 | -35.786557 | 2.05% | 0.27% | motif file (matrix) |
| 2 | 0.903 | G T A C A G T C A G T C G T C A A C G T A T C G C G T A G A C T G A C T A G T C C G T A | 1e-15 | -34.901996 | 2.18% | 0.33% | motif file (matrix) |
| 3 | 0.914 | A G T C A G T C C T G A A C G T A C T G C G T A A G C T G A T C A G T C C G T A C T G A C G A T | 1e-14 | -34.528578 | 1.85% | 0.22% | motif file (matrix) |
